# Monitoring the Response of a Pineal Parenchymal Tumor of Intermediate Differentiation With Cerebrospinal Fluid Dissemination Using a Circulating Tumor Cell Assay

**DOI:** 10.7759/cureus.103982

**Published:** 2026-02-20

**Authors:** Matthew Pelletier, BK Kleinschmidt-Demasters, Perry Corkos, Tony J Pircher, Denise Damek, Santosh Kesari

**Affiliations:** 1 Neuro-Oncology, Pacific Neuroscience Institute, Torrance, USA; 2 Pathology, University of Colorado, Denver, USA; 3 Research, Biocept, Inc., San Diego, USA; 4 Neurology, University of Colorado, Denver, USA; 5 Translational Neurosciences, Pacific Neuroscience Institute, Santa Monica, USA

**Keywords:** circulating tumor cell assay, leptomeningeal metastasis, pineal parenchymal tumor, synaptophysin, tumor cell biomarkers

## Abstract

Pineal parenchymal tumors of intermediate differentiation (PPTID) are aggressive, rare central nervous system tumors. The management of PPTID remains challenging, given the limited number of reported cases and the logistical difficulties in conducting studies on rare tumors such as these. Leptomeningeal disease (LMD) is a relatively frequent pattern of recurrence, and accurate monitoring is essential to optimize potentially efficacious therapies.

We present the case of a 27-year-old woman who initially presented with positional headaches and mild blurry vision. Imaging revealed an enhancing third ventricular lesion with obstructive hydrocephalus. She underwent subtotal surgical resection of the tumor and was diagnosed with PPTID. Approximately 14 months later, imaging demonstrated local recurrence with leptomeningeal metastases.

In addition to standard therapies, the Biocept IN/CNSide cerebrospinal fluid (CSF) liquid biopsy assay (Biocept, Inc., San Diego, California, US), including tumor cell protein expression biomarkers, ctDNA biomarkers, and tumor cell fluorescence in situ hybridization (FISH) biomarkers, was used to monitor treatment response at seven time points. During this period, CSF cytology and other conventional markers were unremarkable.

This case demonstrates how novel CSF-based monitoring technology may improve disease surveillance in patients who develop leptomeningeal disease. Although leptomeningeal metastasis is not uncommon in patients with PPTID, the optimal monitoring approach remains unclear. While this assay remains investigational, it may represent a promising strategy to address an unmet need in the management of PPTID with LMD.

## Introduction

The pineal gland is a small neuroendocrine gland in the epithalamus that plays a role in biological circadian rhythm function and melatonin production. Pineal gland masses are uncommon, comprising less than 1% of all brain tumors [1]. Pineal parenchymal tumors of intermediate differentiation (PPTID) make up a smaller subset of this class of tumors [1]. PPTIDs are considered intermediate-grade pineal parenchymal tumors, WHO grade II or III, depending on mitotic index [2]. Their clinical behavior ranges from indolent to locally aggressive and recurrent disease. Prognosis is influenced by age, extent of resection, and proliferative indices, such as Ki-67 labeling, with five-year survival rates ranging from 50% to 74% [3,4]. Although comprehensive molecular characterization remains limited due to the rarity of these tumors, recurrent alterations, including mutations in KBTBD4, have begun to further refine the biological understanding of pineal parenchymal tumors [2,5]. PPTIDs primarily affect adults, with a median age at diagnosis varying from 30 to 53 years [4,5]. These tumors can cause neurological dysfunction, including headaches, diplopia or visual field loss, and gait dysfunction secondary to obstruction of cerebrospinal fluid, direct invasion, or compression of the surrounding tissue. Up to 62.5% of cases of PPTID can be complicated by leptomeningeal dissemination [4]. As a result of the limited number of reported cases, the optimal therapeutic approaches for these tumors are not well-defined. Gross total resection and radiation are efficacious as a first-line treatment [4]; however, there is an absence of literature pertaining to the management of disseminated disease. Progress in treatment will undoubtedly require a more accurate assessment of the CSF tumor burden. Current guidelines for diagnosis, treatment, and response of leptomeningeal disease (LMD) do not address leptomeningeal metastasis from primary brain tumors [6]. The diagnosis of LMD and assessment of treatment response are often based on a combination of clinical, radiographic, and CSF parameters that may not improve with effective treatment [6-8]. PPTIDs characteristically express synaptophysin, a well-established neuroendocrine marker, making it a biologically rational target for tumor-specific cell-detection assays. To this end, we present a patient with disseminated PPTID treated with systemic and intrathecal therapies and monitored for circulating tumor cells in the CSF using a cell-capture assay that identifies even small numbers of cells via one of the proteins expressed on these tumors, synaptophysin.

## Case presentation

**Figure 1:**
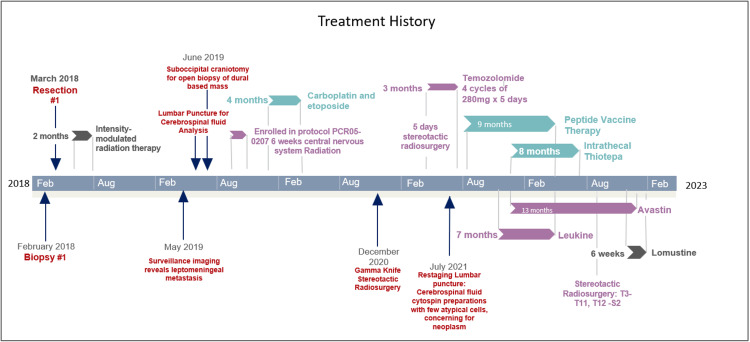
Treatment history Timeline of treatment history, including prior resections, standard therapies, and off-label treatments

**Figure 2:**
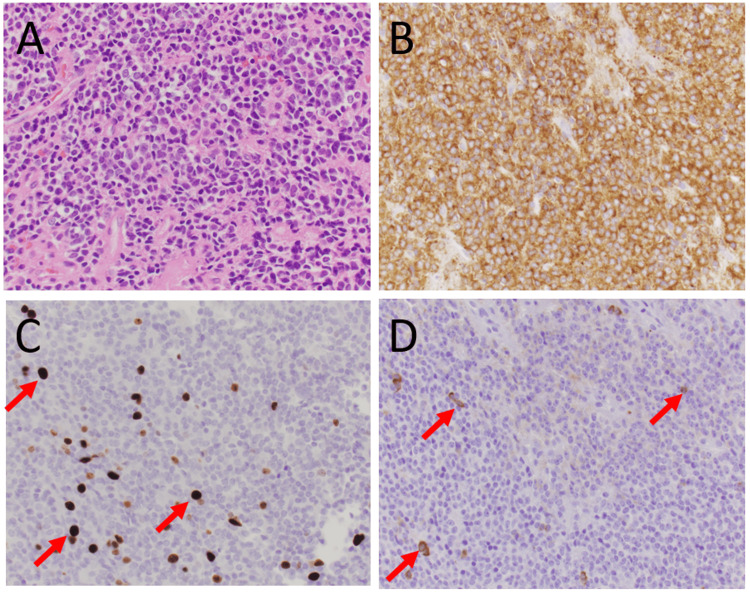
Histology of the tumor Histology of the primary tumor showed a relatively monomorphic small blue cell tumor (A: hematoxylin and eosin staining at 400× magnification), with diffuse synaptophysin immunostaining indicating the neuronal lineage of the tumor (B: synaptophysin immunostaining at 400× magnification). In terms of grading, mitoses numbered 1 per 10 high-power fields, with MIB-1 immunostaining reaching 12% (C: arrows, at 400× magnification). There were scattered cells with neurofilament immunostaining (D: arrows, at 400× magnification).

We present a 27-year-old female who initially presented to her primary care provider endorsing positional headaches and dizziness. MRI brain obtained during initial workup revealed a mass in the third ventricle, resulting in obstructive hydrocephalus. She was immediately treated surgically with third ventriculostomy and biopsy of the lesion (full treatment history summarized in Figure 1). Biopsies showed a relatively monomorphic small blue cell tumor (Figure 2A; at 400x magnification), with diffuse synaptophysin immunostaining indicating the neuronal lineage of the tumor (Figure 2B; at 400x magnification). In terms of grading, mitoses numbered 1/10 high-powered fields with MIB-1 proliferation rate reaching 12% (Figure 2C; at 400x magnification), although there were scattered cells with neurofilament immunostaining (Figure 2D; at 400x magnification). One month later, she underwent a suboccipital craniotomy for subtotal tumor resection. Postoperative MRI confirmed a decrease in the size of the fourth ventricle and residual mass along the septum pellucidum. Following surgical treatment of the lesion, she received 5040 cGy of intensity modulated radiation therapy (IMRT) over 28 fractions. Post-radiation MRI brain demonstrated a complete radiographic response.

**Figure 3:**
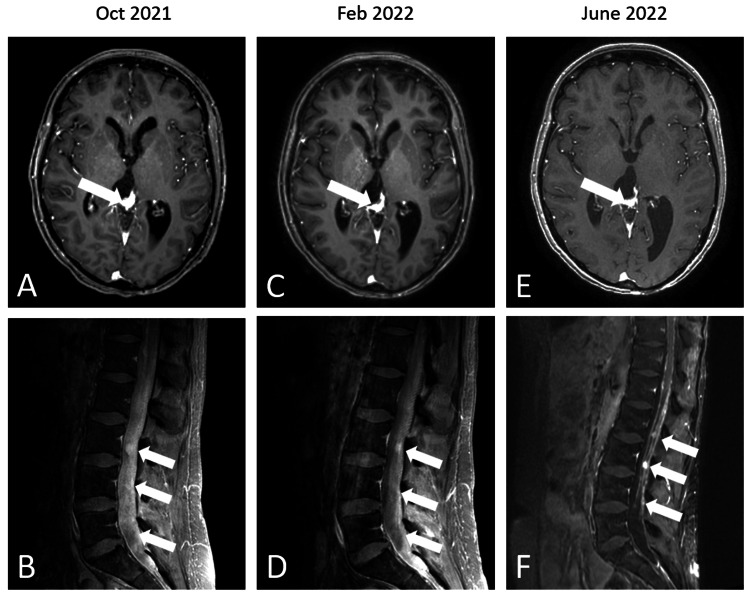
Neuroimaging Axial T1 post-gadolinium imaging of the brain (A, C, E) showing the primary tumor in the pineal region (A) with shrinkage over time (C, E). Sagittal T1 post-gadolinium imaging of the spine (B, D, F) showing extensive leptomeningeal disease throughout the cauda equina, with diffusely thickened and enhancing cauda equina nerve roots and shrinkage over time (D, F).

Surveillance imaging approximately one year later, unfortunately, revealed new bilateral cerebellar lesions. There were no corresponding neurological symptoms or signs. Subsequent investigations and results included: (1) MRI of the whole spine, which demonstrated multiple nodular densities along the spinal cord, most conspicuously from C6-7 through T9, and along the cauda equina at L3-4 (Figure 3); and (2) lumbar puncture showing WBC 3 (69% lymphocytes, 31% monocytes), RBC 6, glucose 44, protein 45, and cytology demonstrating few small lymphocytes and macrophages and a single cohesive group of bland cells favoring meningothelial cells. She was again treated surgically with a suboccipital craniotomy to biopsy the newly discovered cerebellar lesions, and pathology confirmed metastatic PPTID. Soon after, she underwent radiation targeting the whole brain and spine (full treatment summarized in Figure 3). MRI of the brain and spine revealed poor treatment response with increased burden of leptomeningeal disease (LMD). Systemic treatment with intravenous carboplatin and etoposide was administered over six cycles. She responded well and remained without radiographic evidence of LMD recurrence for five months. Surveillance imaging then revealed three discrete subcentimeter enhancing nodules along the left anterior falx and left posterior fossa dura, suspicious for locally recurrent metastatic disease. Three tumors along the anterior falx were treated with stereotactic radiosurgery (SRS). Surveillance imaging one month later showed new lesions within the posterior fossa, with the clinical correlate of a new sixth nerve palsy, prompting fractionated SRS and five cycles of temozolomide and peptide vaccine therapy. She continued to experience diplopia; however, this was corrected with prism glasses, and she was able to continue working and performing activities of daily living.

Surveillance brain and spine MRI after the fifth cycle of temozolomide demonstrated progressive leptomeningeal disease in the thoracic and lumbar spine. There were no corresponding new or progressive neurological symptoms or signs. Cerebrospinal fluid cytology, cell count, glucose, and protein were normal. An Ommaya reservoir was placed, and intrathecal chemotherapy was initiated with triple therapy (cytarabine, methotrexate, and hydrocortisone), but after five doses, the patient developed back pain and left foot drop, with imaging correlating to progressive leptomeningeal disease. CSF cytology and other parameters were normal.

**Figure 4:**
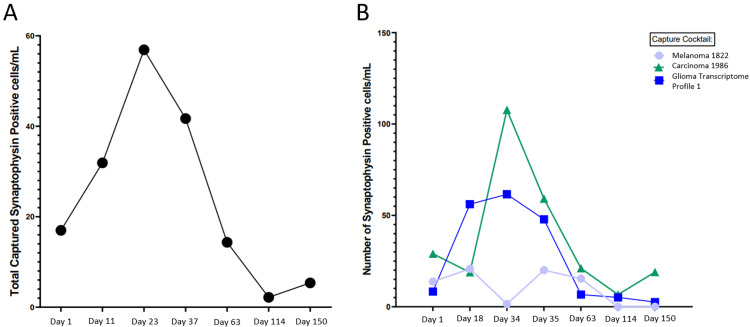
Cerebrospinal fluid collection results A. Total captured synaptophysin-positive cells (cells/mL); B. Total captured synaptophysin-positive cells (cells/mL) using different antibody cocktails for melanoma, carcinoma, and glioma panels.

Intrathecal thiotepa and intravenous bevacizumab were initiated, with additional multi-agent therapy including peptide vaccine therapy, everolimus, osimertinib, sargramostim injections, and 20 cGy proton beam irradiation to bulky disease in the cervical and thoracic spine. The duration and overlap of each therapy are detailed in Figure 1. Additional agents were determined based on biomarker data provided by the Tempus xE panel (Chicago, IL, US), which revealed an EGFR mutation and CCNE1 and MTOR overexpression. The patient sought peptide vaccine therapy independently and was enrolled in NCT04509167. During this eight-month course of therapy, CSF was sent for a Biocept IN/CNSide liquid biopsy (Biocept, Inc., San Diego, California, US), including CSF tumor cell protein expression biomarkers, ctDNA biomarkers, and tumor cell FISH biomarkers; seven specimens were evaluated (Figure 4), in addition to seven non-synchronous CSF evaluations (cytology, cell count, glucose, and protein), serial neurological examinations, and neuro-axis MRI. CSF cytology was negative in all specimens, and other CSF parameters remained unremarkable. Neurological signs and symptoms waxed and waned, with radicular back pain, neuropathic foot pain, intermittent finger paresthesias, and a peripheral seventh nerve palsy that resolved without change in treatment or corresponding MRI finding, possibly representing an unrelated Bell’s palsy. Serial MRIs of the spine revealed decreased disease burden. Cranial imaging did not show evidence of local recurrence.

## Discussion

PPTID are well-known for their variable clinical behavior and significant potential for leptomeningeal dissemination (LMD). Traditional surveillance modalities (surveillance neuroimaging) offer limited sensitivity for early detection of LMD, which significantly impacts prognosis. Our case underscores the limited ability of neurological examinations (Neurological Assessment in Neuro-Oncology-LM or NANO-LM), MR imaging, and CSF cytology to assess LMD [7]. Our case also demonstrates how novel CSF-based diagnostic technologies may enhance disease monitoring and therapeutic decision-making in this challenging clinical context.

The use of a microfluidic, multi-antibody cell capture assay in this case provided quantitative insight into disease burden over time. This platform utilizes a panel of antibodies, including synaptophysin, a neuroendocrine marker strongly expressed in PPTID cells, to quantify circulating tumor cells (CTCs) in the CSF. While standardized performance metrics for CSF CTC assays in PPTID have not been established, studies on other tumor types provide context for their diagnostic utility. In a cohort of patients with breast cancer-associated leptomeningeal disease, the CNSide assay demonstrated a sensitivity of 100% and specificity of 83% relative to traditional clinical criteria [9]. These data suggest enhanced detection capability compared with conventional methods. Unlike standard cytology, which is limited by low sensitivity and reliance on subjective morphological interpretation, this approach allows for objective, quantitative tracking of tumor burden. Furthermore, its non-invasive and repeatable nature enables longitudinal monitoring, offering a potential paradigm shift in the management of CNS tumors with leptomeningeal involvement.

The application of liquid biopsy techniques in CNS malignancies is an emerging area of investigation [10]. CSF offers a more proximal window into CNS pathology. This case builds on early evidence supporting the use of CSF-derived cell-free DNA and CTC-based technologies, expanding their relevance to rare tumors, like PPTID, where standard biomarkers and surveillance strategies are poorly defined [9,11]. This report also contributes to the growing literature emphasizing the high risk of delayed dissemination in PPTID [4]. Multiple case reports have documented late leptomeningeal or dural recurrences, underscoring the need for long-term surveillance and more sensitive monitoring tools. Our findings suggest that microfluidic CTC assays could be particularly valuable in this setting, both for early detection of recurrence and for evaluating response to experimental or salvage therapies.

## Conclusions

PPTID are rare neuroendocrine tumors that are challenging to diagnose and treat. In addition to their complex nature, leptomeningeal spread is not uncommon in these tumors. The importance of craniospinal control in patients with disseminated PPTID has been previously demonstrated. Patients with disseminated PPTID may benefit from the use of synaptophysin capture technology, or other CSF monitoring platforms may serve as adjunctive tools to complement conventional modalities, such as neuroimaging and CSF cytology, in the assessment of leptomeningeal disease. While the current assay is investigational and not yet validated for routine clinical use, this case illustrates its potential role as a supplementary method for disease monitoring. Future studies should explore its diagnostic performance, prognostic significance, and potential integration with next-generation sequencing or molecular profiling to guide personalized therapy.
